# Chemical cues facilitate foraging across the water-land interface in a resident predatory fish

**DOI:** 10.1093/beheco/araf148

**Published:** 2025-12-23

**Authors:** Maya M McElfish, Nicholas A Hess, Helena B Lewis, Sacha E O’Connor, Rita S Mehta

**Affiliations:** Department of Ecology and Evolutionary Biology, Coastal Biology Building, University of California Santa Cruz, 130 McAllister Way, Santa Cruz, CA 95060, United States; Department of Marine Science, Eckerd College, 4200 54th Avenue South, St. Petersburg, FL 33711-4700, United States; Department of Marine Science, Eckerd College, 4200 54th Avenue South, St. Petersburg, FL 33711-4700, United States; Department of Ecology and Evolutionary Biology, Coastal Biology Building, University of California Santa Cruz, 130 McAllister Way, Santa Cruz, CA 95060, United States; Department of Ecology and Evolutionary Biology, Coastal Biology Building, University of California Santa Cruz, 130 McAllister Way, Santa Cruz, CA 95060, United States

**Keywords:** chemosensory, foraging behavior, intertidal, moray eel, land-sea transition, sensory stimuli, multimodal

## Abstract

Transitional ecosystems, such as the land-sea interface, propagate nutrient flow and species interactions. Organisms spanning these boundaries act as important models for understanding the evolution of sensory modalities that promote movement between physically distinct media, and the ecological consequences of ecosystem connectivity. Behavior is fundamentally guided by sensory processing; yet how sensory information is transmitted and collected is heavily dependent on the physical environmental medium. The flow of stimuli across the land-sea interface and the behavioral responses to stimuli are understudied. Vertebrates that span the land-sea boundary offer the opportunity to document how stimuli can be used to complete complex behaviors across transitional ecosystems. We determined that California moray eels (*Gymnothorax mordax*) can use chemical stimuli (odor and taste) to locate prey across intertidal boundaries on Santa Catalina Island. We tested moray responses to chemical stimuli from 4 prey types during high and low tidal conditions, the latter requiring emergence from the water to navigate the land-sea interface. *Gymnothorax mordax* can navigate to a prey source using only chemical stimuli; both when fully submerged underwater and when emerged in the intertidal. Morays showed greater discernment between prey types when exposed in the intertidal zone. When emerged, we observed morays rubbing their faces on the substrate, suggesting odor may be important for detection, with taste further assisting in prey location. This research broadens our understanding of ecosystem connectivity, illustrating how stimuli can cross the land-sea boundary and be used to facilitate predation through a combination of multisensory modalities.

## Teaser

How animals use sensory information across ecosystem boundaries is poorly understood. On Santa Catalina Island, moray eels hunt prey underwater using chemical cues and can follow these cues into the exposed intertidal. Morays were more discriminatory between prey types when they emerged, and face rubbing behavior on land suggests that morays can smell and taste chemical stimuli out of water. Animals that span ecosystems offer insight into how sensory modalities guide predatory behavior across water and land.

## Introduction

Transitional ecosystems provide important connective pathways that allow for temporally dynamic and unpredictable species interactions. Characteristics of the land-sea interface, such as permeable habitat boundaries and high perimeter:area ratios, facilitate the flow and exchange of materials between disparate ecosystems (Marczak et al. 2007; Schlacher et al. 2013). Subsidy transfer and nutrient flows between aquatic and terrestrial systems can be bidirectional, moving from water to land or vice versa (Vannote et al. 1980; Polis et al. 1997; Mellbrand et al. 2011; Gerraty 2023). Yet it remains unclear if and how sensory stimuli move across these boundaries in ways that influence resource use. Effective foraging, especially for unpredictable food resources within dynamic environments, first hinges on successful detection and location of nutrients via the collection and interpretation of environmental sensory information, yet the flow and use of stimuli at the land-sea interface is understudied.

Fish capable of amphibious behavior can act as a model system for the study of water to land transitions in extant and extinct vertebrates (Michel et al. 2015). For instance, aquatic-terrestrial locomotion in extant fishes has been characterized (Pace and Gibb 2009; Pace and Gibb 2011; Pace and Gibb 2014; Standen et al. 2016; Lutek et al. 2022) and has been used to frame hypotheses about evolutionary transitions to terrestrial life (Hsieh 2010; Standen et al. 2016). However, less is known about how sensory systems play a role in the motivation to access the water-land transitional zone. The land-sea boundary offers an opportunity to test how stimuli from food items may move between distinct physical media to facilitate foraging movements across ecosystems.

The rocky intertidal environment offers unique feeding opportunities to predators capable of foraging within this complex landscape. Daily fluctuations in tidal events presumably affect foraging modes as organisms are forced to navigate fully submerged to exposed conditions. Unlike deepwater marine or terrestrial ecosystems, nearshore ecosystems often lack a distinct scavenging guild due to the unreliability and patchy nature of resources that may be scavenged (Britton and Morton 1994). However, the rocky intertidal is an exception, as scavenging opportunities may arise when organisms stranded in tidal pools succumb to drastic abiotic fluctuations over a tidal period (Bridges 1988; Gunderson et al. 2016; Wosnick et al. 2022; Leurs et al. 2023). Active predation or opportunistic scavenging at this ecosystem boundary necessitate stimuli that span 2 distinct physical media, as well as flexibility between sensory modalities as marine predators transition from water to land.

To be accessible to marine predators, stimuli from live prey or carrion must extend from the shore edge or tidal pool into nearshore aquatic habitats, maintaining a distinct trail that a forager could use to navigate across the ecosystem boundary. Not all sensory modes (eg, electro- and mechanosensory modalities) may make the transition between water and air, while certain stimuli (eg, auditory, chemical, visual) are more amenable to transitions between environmental media (MacIver and Finlay 2022). Understanding which stimuli prompt ecosystem-spanning behaviors provides mechanistic context for ecological processes (predator-prey interactions or resource use) and evolutionary patterns, such as selection on sensory modalities that facilitate behaviors across physical media.

Chemical stimuli such as odors or pheromones that emanate outward from a source have strong potential to span the intertidal boundary. Moreover, chemoreceptive structures are highly conserved across vertebrate groups (Whitlock and Palominos 2022), suggesting a shared evolutionary origin for sensory systems. Yet the mechanics of how sensory systems initially designed for underwater use were evolutionarily co-opted for terrestrial life is not fully understood (Nikaido et al. 2013; Mollo et al. 2014 ).The transmission of chemical stimuli as either “odor” or “taste” is dependent on both the molecular structure (eg, in amino acids; Caprio 1978 ) and the media of transport. For example, terrestrial species smell using airborne odorants that are often volatile, hydrophobic in nature whereas taste stimuli are more water-soluble and hydrophilic (Atema 1980; Mollo et al. 2014). This trend is often reversed in aquatic species where odorants tend to be water-soluble and hydrophilic, with gustatory receptors responsive to hydrophobic molecules (Nikaido et al. 2013; Mollo et al. 2014). As such, at the land-sea boundary what may be considered a volatile, hydrophobic, airborne odorant in the terrestrial environment may serve as taste stimuli in the aquatic realm (Mollo et al. 2014). Thus, taste receptors sensitive to hydrophobic molecules in ancestral aquatic organisms are preadapted for transitions into a terrestrial environment, where they can act as odor receptors for hydrophobic molecules (Mollo et al. 2014). Additional evidence for the preadaptive nature of sensory receptors exists at the genomic level; in coelacanths, there are multiple genes encoding receptors characteristic of those in tetrapods for the detection of airborne ligands (Nikaido et al. 2013).

Using such co-opted chemical signals for rapid detection and prey location at the land-sea boundary would be hugely beneficial to an aquatic predator (Laidre and Greggor 2015), yet navigating over extreme shallows and exposed rocks is a high-risk scenario, posing biomechanical and physiological challenges for most large-bodied, fully aquatic, predatory fishes (Heiss et al. 2018). Among aquatic predators, Muraenids (Anguilliformes), collectively known as morays, provide an opportunity to investigate if and how chemical stimuli aid in the detection and location of prey during foraging across tidal contexts at the land-sea interface. Heiss et al. (2018) identified common morphological challenges fully aquatic vertebrates need to overcome in order to forage terrestrially. An elongate, flexible body plan as exhibited by anguilliform fishes circumvents several of these challenges, facilitating some moray species in foraging while emerged from water. Several studies have confirmed moray species moving into the intertidal and navigating between exposed pools to forage (Chave and Randall 1971; Sazima and Sazima 2004; Graham et al. 2009). In the lab, morays similarly exhibit motivation to emerge from the water and overcome a 45° incline to feed terrestrially (Mehta and Donohoe 2021).

The sensory modalities used during the initial stages of foraging (prey detection and location) are understudied for many fish species, including morays. Many predatory fishes are capable not only of using chemical stimuli to locate prey but also of using these cues to make foraging decisions about prey quality and foraging context (Lonnstedt et al. 2012). Chemosensory cues play a particularly important role in foraging for nocturnal species and/or those occupying structurally complex environments (Bryer et al. 2001; Kasumyan 2004; Lonnstedt et al. 2012; Atta 2013). Morays often hunt nocturnally within the confines of reef crevices and thus need to navigate structurally complex habitats with limited light. As such, many moray species would perhaps benefit from chemosensory specialization.

The California moray eel (*Gymnothorax mordax*) is a cryptic, ecologically important predator in nearshore environments off Southern California (Higgins et al. 2018; Mehta et al. 2020). This species exists in high densities, oftentimes residing in shallow (Higgins et al. 2018; Mehta et al. 2020), spatially complex rocky crevices where prey stimuli may be transmitted across a wide range of tidal contexts. Due to their ecology, behavior, and anatomical adaptations, *G. mordax* is an excellent model for studying chemically mediated foraging within the intertidal. Fish (particularly the kelp bass, *Paralabrax clathratus*) make up the majority of prey items *G. mordax* consume, however this species can also consume a wide range of invertebrate prey (Higgins et al. 2018; Higgins and Mehta 2018) found across tidal conditions (eg, crab, lobster, shrimp, and octopus). *Gymnothorax mordax* has been observed feeding opportunistically on bait fishes as well as washed up squid carrion (personal observations). While diet records illustrate the breadth of *G. mordax's* foraging behavior, no studies have investigated the sensory information used to facilitate foraging for this species, and it is unknown if the same sensory cues facilitate foraging across tidal contexts. By testing whether morays use chemical cues across both submerged and intertidal contexts, we can assess if aquatic sensory systems are able to function in transitional environments, offering a framework for hypotheses regarding other extant amphibious fishes and insight into the sensory context faced by early vertebrates during evolutionary water-land transitions.

We conducted 2 summers of field-based behavioral experiments on Santa Catalina Island, California, testing chemical stimuli during both high and low tidal conditions, the latter necessitating that morays partly emerge from the water. We investigated (i) whether *G. mordax* can use chemical stimuli alone to navigate to a specific prey source, (ii) if morays can behaviorally discern between chemical cues from different but ecologically relevant prey species, and (iii) if chemical cues can be used in both fully submerged and partially exposed (intertidal) contexts. Documenting if *G. mordax* can navigate across the water-land transition using only chemical stimuli highlights the capacity for intertidal scavenging by aquatic predators, expanding our understanding of ecosystem connectivity and providing insight into what sensory modalities may be under selection at the land-sea interface.

## Materials and methods

### Study location and sampling sites

**Figure 1:**
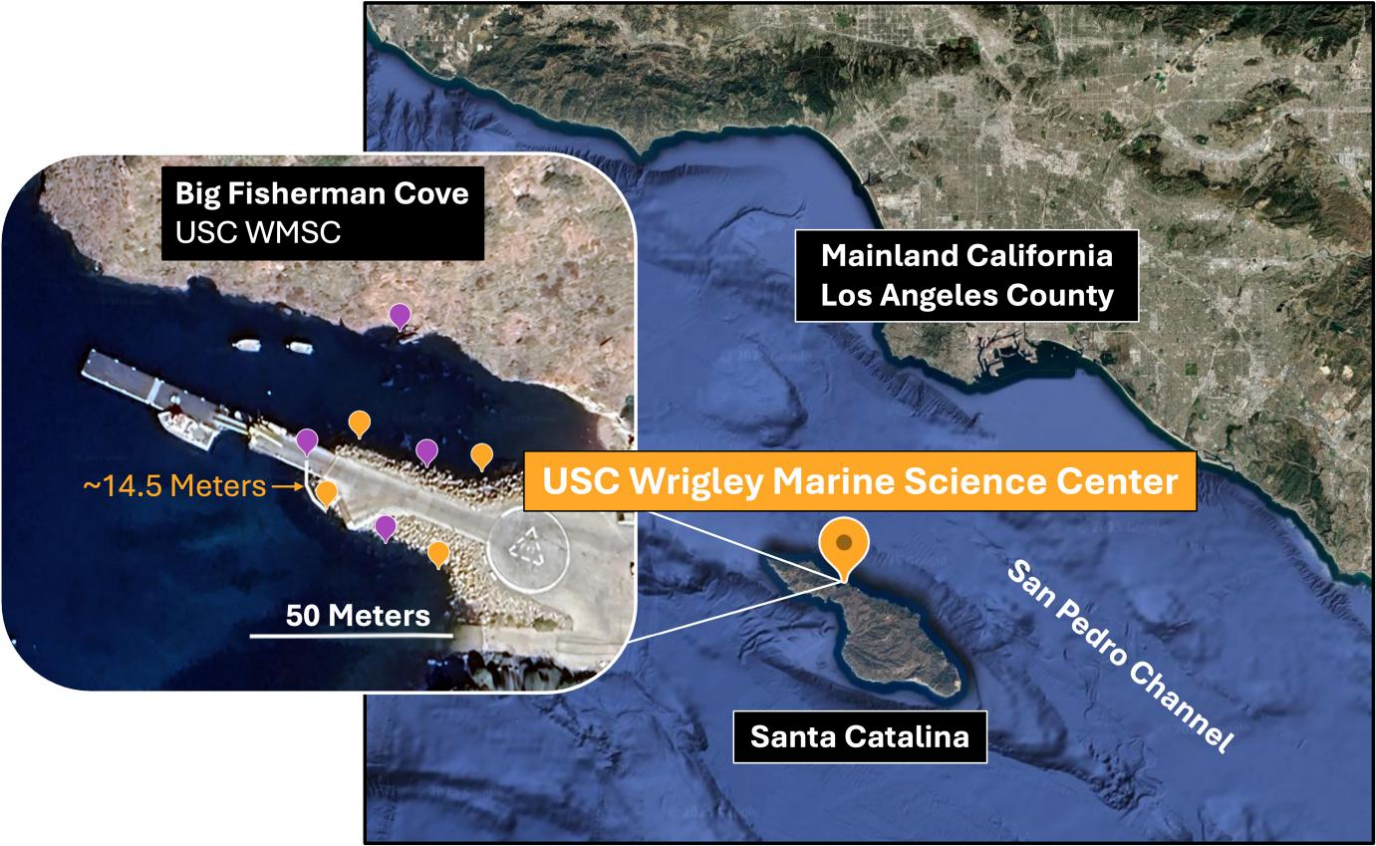
Santa Catalina island and site locations. We tested 8 sites for each treatment at high and low tide for 2 consecutive field seasons. Dots show the approximate site location. Adjacent sites (orange vs purple dots) were at least 10 m apart and never tested on the same day. Map images courtesy of Google Earth 10.95.1.4 (2023), Santa Catalina Island.

This project took place offshore of the University of Southern California's Wrigley Marine Science Center in Big Fisherman Cove on Santa Catalina Island, California (33.4452, 118.4847). We tested 8 sites within the cove (Fig. 1). Sites that were spatially proximal were isolated by physical barriers (eg, pier walls) that prevented individual morays from traversing between sites within the trial timeframe and adjacent sites (Fig. 1, orange versus purple pins) were never tested on the same day. Morays are also known to have high site fidelity so movement between sites, while possible, is unlikely (Mehta et al. 2020). We planned our experimental design according to the tides to test each site at high and low tide to observe morays in a fully submerged context (> 1 m of water) and a partially exposed context (< 5 cm of water), which required morays to emerge from the water with their bodies partly exposed to navigate rocky substrate.

### Chemical stimuli

We tested chemical stimuli from 4 potential prey species; kelp bass (*Paralabrax clathratus*), 2-spot octopus (*Octopus bimaculoides*), both regularly found in stomach contents (Higgins et al. 2018), and anchovy (*Engraulis sp.*), squid (*Doryteuthis opalescens*) which opportunistically appear in the moray diet (Mehta et al. 2020). To prepare cues, we emulsified whole prey (4–6 individuals) in 500 mL of 50 μm filtered seawater for 30 s. Because prey odors are composed of species-specific complex chemical mixtures (Atema 1980), using whole prey ensured the treatments are inclusive to the full array of chemical odorants that may emanate from each prey item. The emulsified solution was strained through a 1.0 mm sieve to remove particulate matter and was subsequently diluted with 50 μm of filtered seawater such that the final solution contained 0.05 g of tissue per 1 mL of 50 μm filtered seawater (modified from methods found in Dixson et al. 2015; Gervais and Brown 2021, which test chemical cue concentrations of 0.5 g and 0.04 g tissue per 1 mL, respectively). Testing 0.05 g tissue per 1 mL seawater reflects a concentration on the lower end of the spectrum reported in the literature (eg, Gervais and Brown 2021), helping ensure our treatments were within the realm of environmentally realistic stimuli that may emanate outward from a carrion or injured prey source. For cephalopod cues, we removed ink sacs prior to blending to prevent enhanced coloring from the ink, and for compounds in ink acting as a deterrent/chemosensory disruptor (Derby 2014). For larger kelp bass specimens, heads and caudal fins were removed to facilitate blending. The control treatment consisted of 50 μm filtered seawater (as per Gardiner and Atema 2010; Lonnstedt et al. 2012), which was also blended and strained, and then further diluted with filtered seawater to mimic the processing of the prey treatments, and served to control for the flow of liquid entering the experimental site. All treatments were frozen until use (maximum 14 days of storage).

### Trial format and experimental apparatus

**Figure 2:**
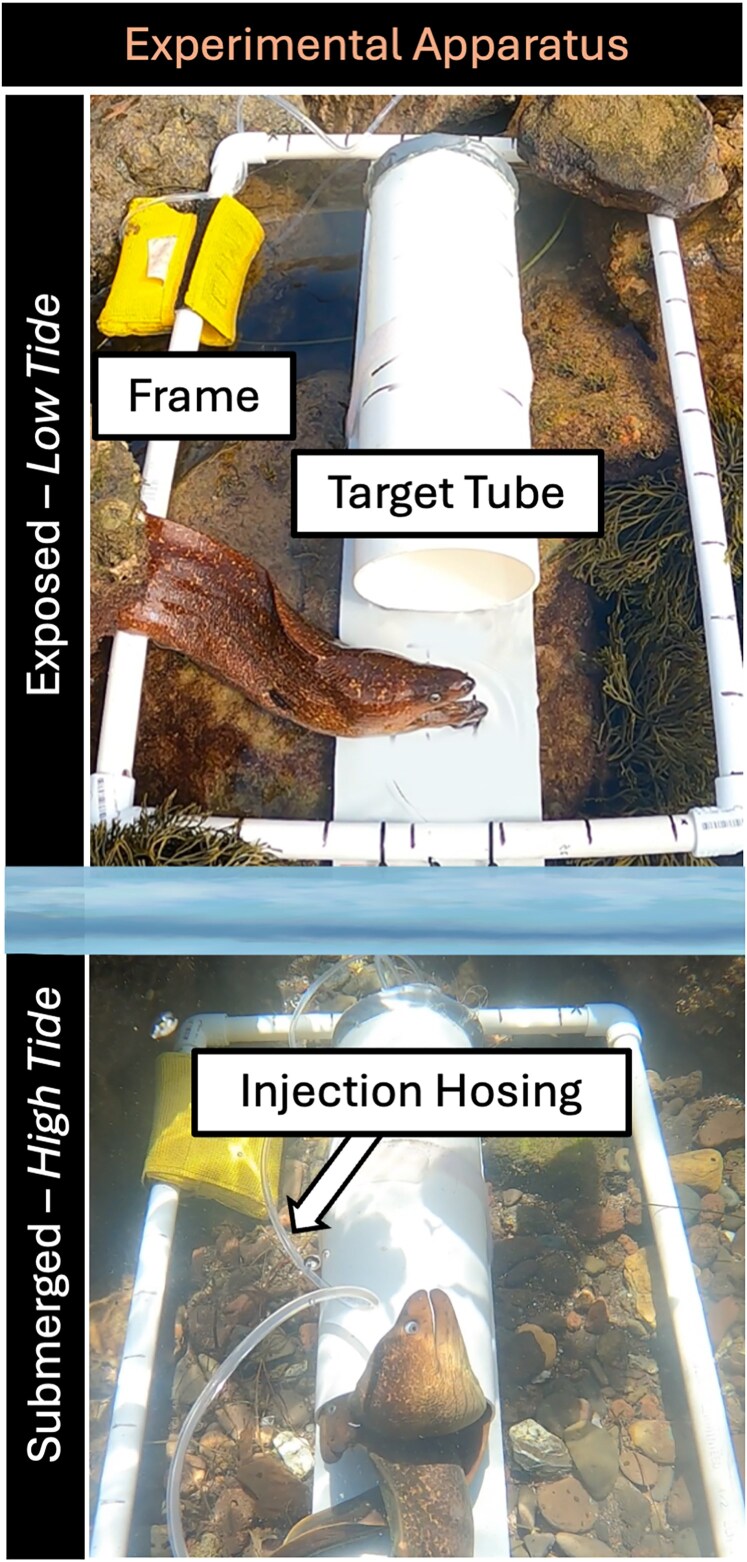
Experimental design. The experimental apparatus consisted of a camera mounted to a 1,500cm^2^ Frame, which surrounded a central PVC Target Tube. Data was collected with respect to both the Frame and the Target. Treatments were injected into the target from the surface using vinyl injection hosing.

Our experimental apparatus was designed to administer controlled pulses of chemical stimuli into a target area within our sample site. The experimental apparatus was fixed with an underwater camera (GoPro Hero 8), recording the entire duration of the trial. Treatments were injected to the site via vinyl tubing (4 mm diameter) into a 30 cm long, 10 cm diameter white PVC tube (Fig. 2). The PVC tube acted as a “foraging crevice” (mimicking rocky crevices in the habitat) and is henceforth referred to as the “target”. The target required morays to navigate to a specific area from where the cue was emanating. We limited our observations to a 1,500 cm^2^ framed area (50 × 30 cm) around the target tube (Fig. 2), which was denoted physically by a PVC edge (2023 trials) or measured out within video footage (2022 trials). Trials lasted 15 min, with 100 mL of the treatment injected in the first minute, and 30 mL injected every 2 min with the last injection occurring at minute 13, at which point the vinyl injection hosing was flushed with seawater. Mock trials with dye prior to experimentation confirmed that injections directionally exited the target in a reliable and predictable way.

Each of the 4 prey treatments and the control were administered at both tidal levels for each site, with treatment order being randomized. We opted for a randomized design to administer treatments over a prepost design, as the later assumes environmental stability between treatment applications, which was not guaranteed in the dynamic nearshore environment. A site would only be administered 1 of the 4 prey treatments on a given day, either at high or low tide, and was not revisited for at least 48 h. Because *G. mordax* exhibits high site fidelity (Higgins and Mehta 2018), repeatedly testing sites in this manner allowed us to present the same eels with the treatments. We strived to test each site (*n* = 8) at both tidal levels (submerged and exposed) for each of the 5 treatments. Thus, each site was visited a total of 10 times throughout an 8-week field season. We repeated these procedures over 2 consecutive field seasons (2022 and 2023), using the same 8 sites. As such, for each treatment and tidal condition combination (eg, control:submerged or anchovy:exposed) we have 8 trials per summer, and up to 16 total trials across 2 summers.

**Figure 3:**
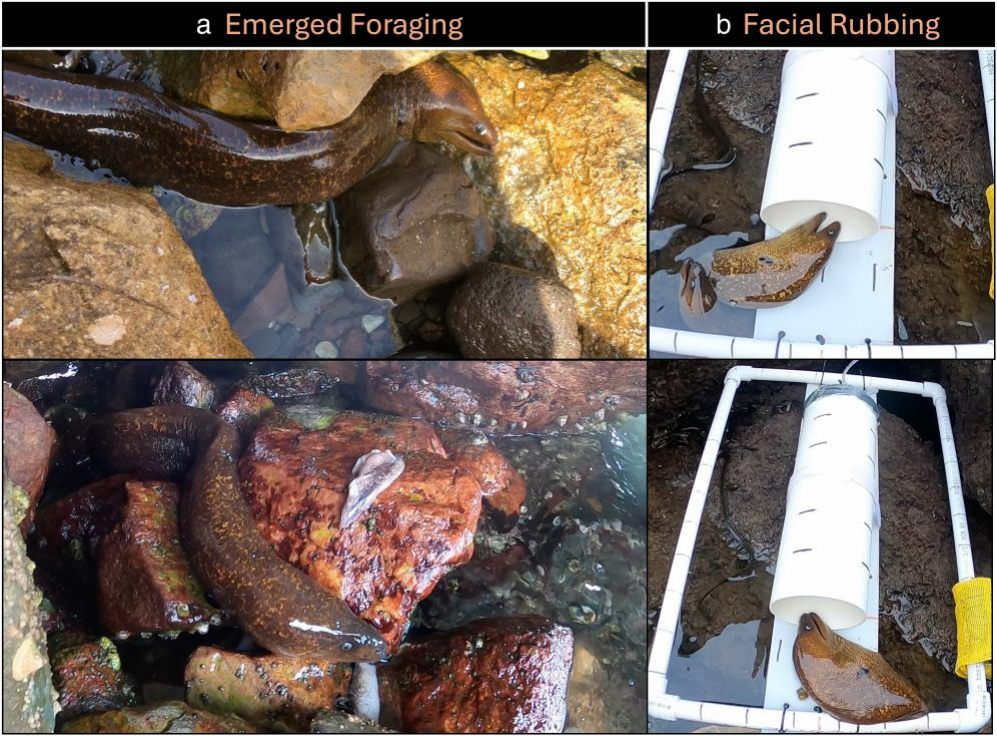
Behavioral observations. (a) Nearly full emergence from the water to feed (b) Observations of morays rubbing their faces along the substrate.

Lastly, we were interested to document if *G. mordax* could complete their full foraging sequence during an emerged state. After all trials were completed, we placed squid bait within the rocky intertidal, and recorded if morays could emerge, locate, and fully consume prey out of water. However, because we did not want to repeatedly provision morays and alter their foraging behaviors at sites that have a long history of supporting a variety of research programs; these observations were limited to 5 recorded feeding events during year 2. Multiple morays emerged, navigated to the bait, and successfully consumed the prey, confirming that these stimuli and modalities can be used to complete an entire feeding cycle out of the water (Fig. 3a, Supplementary Materials).

We also observed morays rubbing their faces, specifically the side of their jaw, along the substrate. We observed the moray position its jaw so that it made contact with the substrate around the target entrance and informally observed this behavior more often in the exposed condition where morays emerge (Fig. 3b and Supplementary Materials). Face rubbing could not be properly quantified because if morays fully entered the target, views of facial rubbing were obscured. Nonetheless, this behavioral observation is insightful because morays have high densities of taste and touch receptors along this region of the body (Bardach et al. 1959; Bardach and Loewenthal 1961).

### Summary of key terms

We refer to the PVC tube itself as the “target” because this is the release site of the chemical information. The immediate 1500 cm^2^ vicinity around the target is referred to as the “frame”. Treatments refer to any of the injected liquids, with “control” referring to seawater and “stimuli” referring to any of the 4 prey treatments. “Submerged” refers to trials occurring fully underwater, while “exposed” refers to trials occurring at the land-sea interface, requiring morays to at least partially emerge from the water.

### Video analysis

To analyze trial footage we used an ethogram coding program (BORIS—Friard and Gamba 2016). We tallied (yes/no) if an eel entered the frame or target. An individual was considered to have “entered” the frame or target the moment their eye passed the frame edge or target entrance, as apparent chemosensory organs are anterior to the eye in morays. We documented the total duration at least 1 moray was present within the target (“Target Duration”), and the maximum number of individuals within the target observed at 1 time. For duration data, an individual was considered to have “exited” the target after the complete removal of the face, from the eye to the end of the rostrum. For exposed trials, the percent emergence (ie, the amount of body out of water) was not quantified because the moray's elongated morphology prevented the entire body of the individual from being within the field of view of the camera. However, we estimate anywhere from 30% to 80% emergence, with the anterior third of the body often observed out of water.

### Data analysis

RStudio (Version 2024.12.0 + 467) was used for statistical analysis. We analyzed all data using Generalized Linear Mixed Effects Models (GLMM), with the glmer function from the lme4 package (Bates et al. 2015). This allowed us include “site” (ie, the experimental location) and “summer” (2022 and 2023) as random effects when the addition of these parameters lowered the AIC value.

To report differences between the control and prey treatments for trials and target visitation, we modeled the data using a GLMM with a binomial distribution. To compare the duration spent within the target we used a GLMM with a gamma distribution and log link function. If no eels were present within the trial (ie, no eels observed within the framed area), the duration inside the target was logged as “NA”. If eels were present within the frame but failed to find the target, the duration was logged as 0 s in the target. We differentiated between these scenarios because we were interested in tracking the duration in the target when eels were fully known to be within the area. In cases where zero values were present in the data, a small constant value of 0.1 was added to avoid violating the assumptions of gamma distributions. For the control treatment we observed 2 morays in the submerged context and 1 moray in the exposed context throughout the 2 summers. As such, duration data for the control was deficient (eg, composed of NA's for trials without eels) and incompatible in our analysis. We excluded the control from this comparison to instead focus on the differences between the 4 prey treatments.

### Ethical note

This research was conducted on Santa Catalina Island following California Department of Fish and Wildlife guidelines (Permit 190830002-19986-001). Morays were not handled or harmed during this experiment.

## Results

### Percent visitation

**Figure 4:**
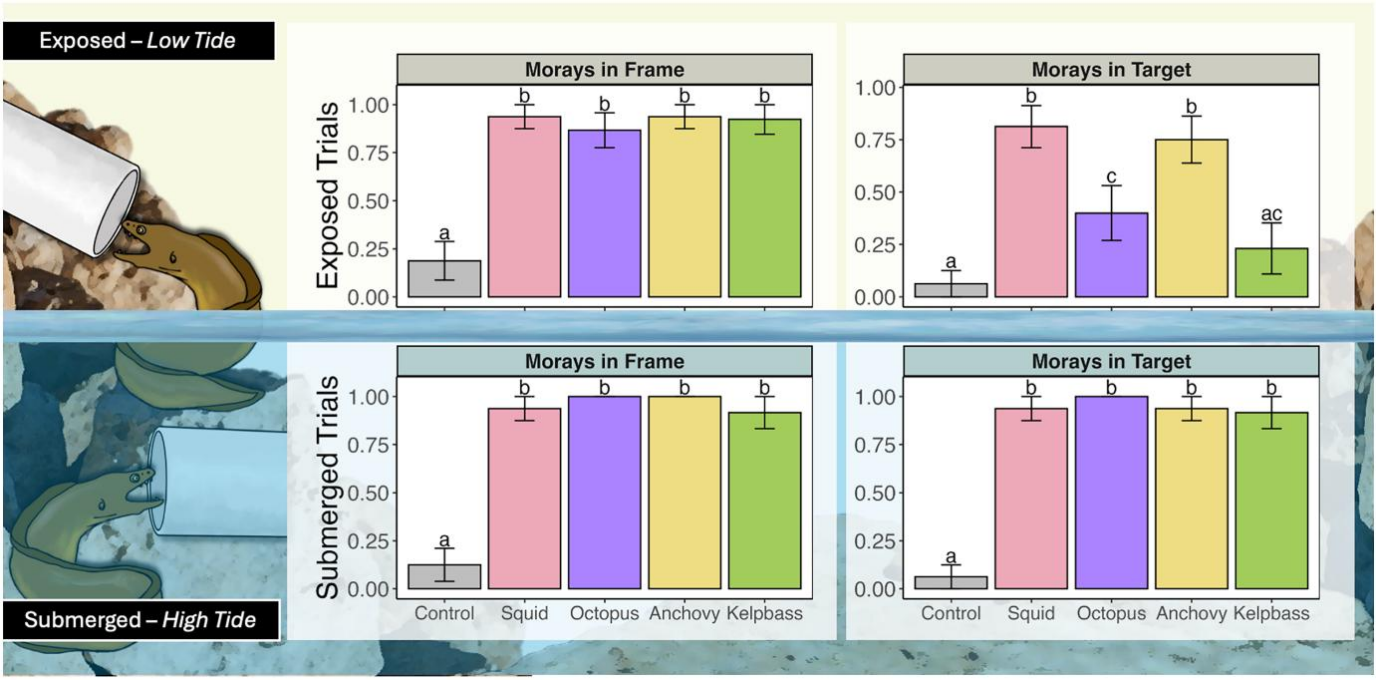
Frame and target visitation. Graphs depict the proportion of trials where morays were observed in the trial vicinity (ie, within the frame—leftmost graphics) and observed entering the target (rightmost graphics). The top panel shows results for exposed tidal conditions, and the bottom panel shows submerged conditions. Bars are the proportion visitation per treatment ± standard error. Letters denote significant differences between treatments within the plot.

We compared the percent visitation (Fig. 4) to the trial vicinity (eg framed area) and percent visitation to the target (eg, tube). Our goal was to test each site for all treatments at both tidal conditions, however a few trials experienced human disturbances (eg, emergency helicopter landing) and were excluded from analysis. As such, replicates range from 12 to 16 trials per tidal condition.

**Table 1:** Percent visitation.

Exposed—frame visitation				
Predictor	Estimate	SE	*z*-value	*P*-value
**(Intercept) Control**	−1.655	0.849	−1.950	0.051
**Treatment Anchovy**	4.626	1.487	3.112	0.002
**Treatment Kelp Bass**	4.326	1.457	2.970	0.003
**Treatment Octopus**	3.675	1.196	3.072	0.002
**Treatment Squid**	4.626	1.487	3.112	0.002

Exposed—Target visitation				
Predictor	Estimate	SE	*z*-value	*P*-value
**(Intercept) Control**	−2.993	1.134	−2.639	0.008
**Treatment Anchovy**	4.253	1.303	3.264	0.001
**Treatment Kelp Bass**	1.542	1.267	1.217	0.224
**Treatment Octopus**	2.499	1.219	2.050	0.040
**Treatment Squid**	4.658	1.345	3.463	0.001

Submerged—Frame visitation				
Predictor	Estimate	SE	*z*-value	*P*-value
**(Intercept) Control**	−5.781	3.381	−1.71	0.087
**Treatment Anchovy**	—	—	—	^a^
**Treatment Kelp Bass**	12.066	5.709	2.113	0.035
**Treatment Octopus**	—	—	—	^a^
**Treatment Squid**	13.908	5.651	2.461	0.014

Submerged—Target visitation				
Predictor	Estimate	SE	*z*-value	*P*-value
**(Intercept) Control**	−3.723	1.563	−2.381	0.017
**Treatment Anchovy**	7.481	2.033	3.679	< 0.001
**Treatment Kelp Bass**	7.028	2.020	3.478	0.001
**Treatment Octopus**	—	—	—	^a^
**Treatment Squid**	7.481	2.033	3.679	0.0002

Model output (GLMM for binomial data with logit link function) for the percent of trials morays visited the frame and target in both exposed and submerged conditions.

^a^Model could not estimate effect due to complete separation (100% visitation); estimate and *P*-value not reliable.

### Within frame

Eels were observed entering the framed area across all prey treatments and tidal conditions, (Fig. 4, left panels). This ranged from 100% visitation in the submerged context for anchovy (*N* = 16) and octopus (*N* = 12) treatments, to 86% visitation in the exposed context for octopus (*N* = 15). In contrast, < ∼18% of control trials (*N* = 16) were visited. There were no significant differences between prey treatments in either tidal condition, and all prey treatments were significantly different than the control treatments (*P* < 0.001 for all exposed prey: control contrasts, and *P* = 0.01 for submerged contrasts, Table 1).

### Within target

Morays were able to find the target (Fig. 4, right panels) within the frame for all prey treatments in the submerged context compared with the control, with no significant differences between prey treatments. In the exposed tidal condition, morays entered the target significantly more for squid (percent ± SE = 0.81 ± 0.10, *P* = 0.001), octopus (percent ± SE = 0.4 ± 1.3, *P* = 0.04), and anchovy (percent ± SE = 0.75 ± 0.11, *P* = 0.001) treatments compared with the control (percent ± SE = 0.063 ± 0.063, Table 1). Between prey treatments, there was significant variation in target visitation for the exposed tidal context, with eels visiting the squid more than kelp bass (percent ± SE = 0.23 ± 0.12, *P* = 0.003) and octopus (*P* = 0.02) as well more visits to the anchovy than kelp bass (*P* = 0.007) and octopus treatments (*P* = 0.04).

### Duration in target

**Figure 5:**
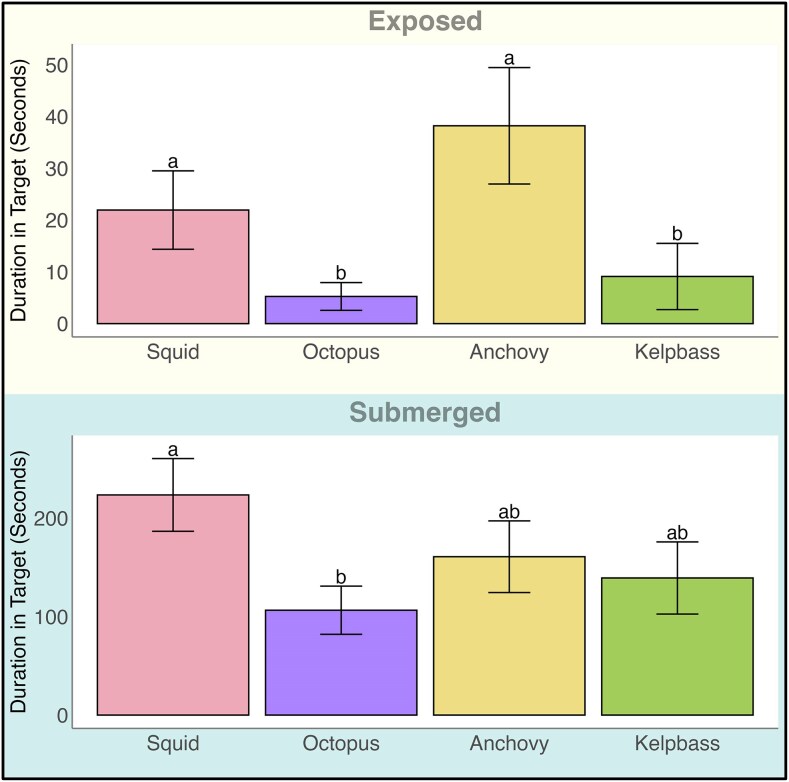
Duration in target. Bars depict time in seconds (±standard error) spent within the target tube for each prey treatment. Letters denote significant differences between treatments within the plot. For the exposed condition, there were multiple significant differences between treatments—Anchovy: Kelp Bass, Anchovy: Octopus, and Squid: Octopus (*P* < 0.01), and Squid: Kelp Bass (0.04). Control was excluded from graphics due to low sample sizes.

When morays appeared in a trial, we compared the average duration spent within the target across prey treatments. In the exposed context, there was no difference between squid and anchovy treatments or kelp bass and octopus treatments (Fig. 5, Table 2). However, morays spent significantly longer durations in the target for squid (mean ± SE = 21.93 s ± 7.57 s) and anchovy (mean ± SE = 38.4 s ± 11.24 s) when compared with octopus (mean ± SE = 5.24 s ±2.68 s) or kelp bass (mean ± SE = 9.01 ± 6.39). In the submerged context, no prey treatment contrasts were significant for duration except between squid (mean ± SE = 223.46 s ± 36.92) and octopus (mean ± SE = 106.43 s ± 24.46, *P* = 0.01).

**Table 2:** Duration in target.

Exposed				
Predictor	Estimate	SE	*z*-value	*P*-value
**(Intercept) Anchovy**	3.684	0.478	7.700	1.36E−14
**Treatment Kelp Bass**	−2.313	0.883	−2.619	0.009
**Treatment Octopus**	−2.872	0.860	−3.341	0.001
**Treatment Squid**	−0.585	0.595	−0.983	0.326

Submerged				
Predictor	Estimate	SE	*z*-value	*P*-value
**(Intercept) Anchovy**	4.799	0.322	14.918	2.51E−50
**Treatment Kelp Bass**	−0.334	0.300	−1.114	0.265
**Treatment Octopus**	−0.154	0.315	−0.488	0.626
**Treatment Squid**	0.448	0.287	1.558	0.119

Model output (GLMM for a gamma distribution, and log link function) comparing the duration spent within the target for each prey stimulus, when eels were observed in the frame.

### Number of observed individuals

For all treatments, about twice as many individuals were observed in the submerged context compared with the exposed context (Table 3). Squid and anchovy treatments had the highest total sum of individuals (15 each in Exposed and 29 and 31, respectively, in Submerged).

**Table 3:** Number of morays observed per treatment and tidal condition, on average across all trials as well as the sum total number observed over all trials.

Treatment	Exposed		Submerged	
	Mean ± SE	Total Morays Observed	Mean ± SE	Total Morays Observed
**Control**	0.06 ± 0.06	1	0.13 ± 0.09	2
**Anchovy**	0.94 ± 0.19	15	1.94 ± 0.27	31
**Kelp Bass**	0.38 ± 0.18	5	1.42 ± 0.19	17
**Octopus**	0.40 ± 0.13	6	1.42 ± 0.23	17
**Squid**	0.94 ± 0.14	15	1.81 ± 0.28	29

## Discussion

Foraging is a deliberate process composed of sequential behavioral steps, beginning with detection and location of a food resource, followed by capture and/or handling, and concluding with ingestion (Wootton et al. 2023). Each of these behavioral steps is dependent on sensory processing (Vickers 2000; DeBose and Nevitt 2008). In this study, morays were more responsive to all prey treatments in terms of trial and target visitation compared with the control (where we only observed 3 morays over 2 sampling years). We demonstrate that *G. mordax* can use trace chemical information from whole prey (0.05 g tissue:1 mL water) to navigate within the vicinity of the cue (ie, within the framed area) equally well regardless of both prey treatment and tidal condition. The ability to use these cues in both submerged and exposed contexts demonstrates that chemical stimuli can span this ecosystem boundary, and sensory function in morays persists when moving across the land-sea interface.

For the fully submerged tidal context (> 1 m depth) morays were successful in locating the target, regardless of prey treatment, and the total duration spent within the target was not significantly different between squid, anchovy, and kelp bass. The behavioral responsiveness and overall success in target location in these fully submerged trials illustrates that chemical stimuli play an important role for morays searching for prey underwater.

In the exposed tidal conditions, we found that *G. mordax* uses chemical stimuli to successfully locate a target that is out of the water. However, morays visited the target less often for kelp bass and octopus in the exposed condition compared with other treatments. Trends from the submerged context appear heightened in exposed trials, with longer durations in the target for squid and anchovy, and with reduced activity in response to octopus and kelp bass treatments. This might be reflective of ecological tradeoffs, where nutritional benefits of the prey (Kohl et al. 2015) may need to outweigh the costs of navigating into exposed areas. Exposure is potentially dangerous (due to stranding, desiccation, or terrestrial predators), and navigating onto land by overcoming an incline may be energetically demanding to accomplish. Research examining the caloric content of prey used in this study could help support a cost/benefit hypothesis. Alternatively, the chemical composition (hydrophobic vs hydrophilic molecular composition) of these “preferred” prey items may be more conducive to tracking their dispersal pattern from the source or are more easily followed across the semi-terrestrial barrier. Further identification of the components (eg, amino acids, lipids) of these items, and which of these components are used during prey detection/location, would be an interesting area of future research. This would provide a mechanistic understanding of how fish integrate chemical information across different physical media to guide behavior, offering insight into how the senses are used in extant amphibious taxa, or were used during evolutionary transitions from water to land.

With respect to the sensory modalities in this study, we documented morays rubbing their faces along the substrate or target entrance. We define face rubbing as a behavior where morays sweep the lateral edge of the lower jaw along the substrate. We believe this is indicative of a more tactile or gustatory modality, as morays have high densities of gustatory cells along their jaw (Bardach et al. 1959) while touch corpuscles are densest on the lips and the apex of the snout (Bardach and Loewenthal 1961). Terrestrial odorants are typically hydrophobic, yet for aquatic species these same molecules are associated with the modality of taste (Mollo et al. 2014). We hypothesize that morays may use odor from hydrophilic molecules diffusing into the water and then employ taste during face rubbing behavior with hydrophobic molecules, especially while emerged to continue following the chemical trail into the target. The multimodal nature of using both odor and taste during distinct phases of foraging is consistent with observations of other moray species. Bardach et al. (1959) demonstrated that olfactory cues seem to be used to detect and generally locate a prey item, but subsequent activation of taste and touch with physical prey were required to initiate a feeding response in *Gymnothorax moringa* and *G. vicinus* while submerged.

Lastly, comparing the sum totals of observed eels there were roughly half the number of individuals in exposed trials compared with submerged trials. This suggests that the transition into the shallows may be a barrier for some individuals, or that transitioning into this area has extended costs that not all individuals can support. One explanation for this observation may be biomechanical; perhaps certain size classes lack the strength or flexibility to navigate while emerged over the rocky substrate (Gans 1962; Mehta et al. 2021). Although we could not get a reliable estimate of body size from the footage to verify this hypothesis, an individual's size has been shown to play a role for other fish species making use of this transition zone. It was observed that European catfishes hunting for pigeons at the waterside, and moving up onto land to capture prey tended to represent the smaller range of individuals inhabiting a study area (Cucherousset et al. 2012). Contrary to these findings, larger Canterbury mudfish were found to migrate terrestrially to escape hypoxic conditions whereas smaller fish stayed in hypoxic waters (O’Brien 2007). Both findings support the idea that size may influence movement across transition zones. Alternatively, an ecological hypothesis might be related to individual hunger/nutrient demands increasing risky foraging (Toscano et al. 2016), and/or that there are individual personality differences (eg, with respect to boldness) influencing an individual's capacity for risk-prone behaviors (Toscano et al. 2016). The observed reduction in morays during the exposed condition compared with the submerged emphasizes that there is a facet of variability (be it personality, ecological, or biomechanical) within the population that may serve as a basis for natural selection.

The capacity to use trace chemical information from prey (either as odor or taste) to precisely locate a target illustrates that *G. mordax* is well suited to take advantage of opportunistic foraging or scavenging resources that are inaccessible to most other predatory bony fishes within the nearshore community off Southern California. Furthermore, the responsiveness of morays to these cues suggests that chemical information plays an important role in their natural history and foraging ecology, regardless of depth. The tides can reorganize prey distribution and abundance in ways that predictably guide predator-prey interactions, exposing the abilities of predators that can move through physically different habitats such as water and land. Relatively large-bodied, aquatic, predators hunting across tidal contexts inspire a series of interesting and ecologically important questions related to trophic linkages, ecosystem connectivity, and nutrient flow. Although intertidal foraging opportunities are patchy, they are not unprecedented. At this study location, we have observed large schools of bait fishes like anchovies trapped in shallow sheltered bays when chased by predators, pelagic squid washed up the intertidal, and octopus using small tidal pools to forage. Our findings highlight that these cues span the ecosystem boundary and that *G. mordax* is poised to take advantage of potential food resources in the intertidal, as evidenced by their readiness to emerge for prey chemical information disseminating from a source.

Such movements from water to land are also pivotal with respect to vertebrate evolution, and studying extant animals capable of making these transitions helps lend insight into physiological and morphological adaptations that facilitate navigating physically distinct environmental media. Because both the stimuli and cell receptors associated with smell and taste are highly dependent on the environmental media for dissipation and collection, assessing the functionality of both stimuli and sensory modalities at this transition zone provides context for the behavioral motivation to span the aquatic-terrestrial interface. When aquatic predators, like moray eels, follow cues across the water-land boundary, it offers a window into the sensory and locomotor adaptions which may have facilitated early evolutionary transitions to terrestrial life.

This ecological flexibility is not unique to *G. mordax*. There are over 200 species within Muraenidae, with some species exceptionally amphibious in nature, and capable of crossing semi-terrestrially between patchy tidal habitats (Chave and Randall 1971; Sazima and Sazima 2004; Graham et al. 2009). The elongated body plan in muraenids facilitates spatially confined crevice predation (McElfish et al. 2024) and also assists with movement (Heiss et al. 2018; Mehta et al. 2021) into the intertidal and shallow pools (Chave and Randall 1971; Graham et al. 2009). Not only can *G. mordax* (and other moray species) detect chemical information, but their elongate body plan further allows them to physically navigate over extreme shallows and physical barriers in a controlled and coordinated way, in contrast to other predatory fish. These morphological traits, now paired with our results demonstrating that chemical information can connect disparate habitats, highlights how both anatomy and sensory processing contribute to foraging movements across contrasting environments. The ecological diversity with the muraenid clade alone (from fully aquatic species to more intertidal species) can be leveraged to comparatively examine how sensory and locomotor traits have evolved to facilitate movements across aquatic-terrestrial gradients.

This study is the first to document that an important predator in southern rocky reef systems of California, the California moray, can use trace chemical information to rapidly locate food resources in both fully submerged and exposed contexts. This study also provides a way to test if chemical information can span the land-sea boundary to facilitate foraging by fully aquatic fish predators. We advocate for more research focused on animal foraging at the land-sea interface to further merge current ecological and macro evolutionary perspectives.

## Supplementary Material

araf148_Supplementary_Data

## Data Availability

Analyses reported in this article can be reproduced using the data provided by McElfish et al. 2025.
